# Green Surgery: Current Evidence, Challenges, and Future Directions

**DOI:** 10.3390/ijerph23081012

**Published:** 2026-08-02

**Authors:** Aikaterini Mastoraki, Maximos Frountzas, Foivos-Konstantinos Stamatis, Maria Batagianni, Emmanouil I. Kapetanakis, Panagiotis Sakarellos, Paraskevas Stamopoulos, Napoleon Moulavasilis, Evangelos Fragkiadis, Dimitrios Schizas

**Affiliations:** 1 First Department of Surgery, “Laikon” General Hospital, National and Kapodistrian University of Athens, 11527 Athens, Greece; fstamatis02@gmail.com (F.-K.S.); mrbatagianni@gmail.com (M.B.); panagiotissakarellos@gmail.com (P.S.); schizasad@gmail.com (D.S.); 2 Department of Colorectal Surgery, Royal Marsden NHS Foundation Trust, London SW3 6JJ, UK; froumax@hotmail.com; 3 Department of Thoracic Surgery, “Attikon” University Hospital, National and Kapodistrian University of Athens, 12461 Athens, Greece; emmanouil.kapetanakis@gmail.com; 4 Department of Surgery, General and Oncologic Hospital of Kifissia ‘Agii Anargiri’, National and Kapodistrian University of Athens, 14564 Athens, Greece; pstamop@gmail.com; 5 First Department of Urology, “Laikon” General Hospital, National and Kapodistrian University of Athens, 11527 Athens, Greece; napomoul@hotmail.com (N.M.); e.fragkiadis@gmail.com (E.F.)

**Keywords:** green surgery, OR sustainability, clinical practice, future perspective

## Abstract

**Highlights:**

**Public health relevance—How does this work relate to a public health issue?**

- The operating room is a major contributor to healthcare-associated carbon emissions, being responsible for a disproportionate share of hospital energy use, single-use item waste, and resource consumption.
- Green Surgery as a concept addresses the healthcare sector’s role in climate change by promoting eco-friendly practices in surgical care, directly linking clinical decision-making with environmental responsibility.

**Public health significance—Why is this work of significance to public health?**

- Evidence from leading hospitals worldwide shows that sustainable surgical practices—including waste reduction, reusable equipment, and low-impact anesthetic agents—can significantly reduce carbon emissions without compromising patient outcomes.
- Achieving net-zero surgical care requires multidisciplinary collaboration across surgical teams, hospital management, and policymakers, reinforcing that environmental sustainability constitutes a collective public health priority.

**Public health implications—What are the key implications or messages for practitioners, policymakers and/or researchers in public health?**

- Surgical teams and hospital executives should endorse the 10R circular economy model as a practical framework for designing institution-specific green initiatives and measuring their environmental impact.
- Policymakers and surgical societies should prioritize standardized sustainability metrics, education on carbon hotspots in the OR, and incentives for the adoption of Life Cycle Assessment in surgical research.

**Abstract:**

Climate change has been identified by the World Health Organization (WHO) as “the single greatest health threat to humanity”. To investigate this subject, we conducted a narrative review of the literature about sustainability in the OR, with the aim of exploring the existing knowledge and identifying prevailing challenges in the field. A combined automated and manual database search of the literature was performed using Medline (PubMed), Scopus, Ovid, and the Cochrane Library, covering publications available up to and including 21 May 2025. The significance of environmental sustainability is recognized across all healthcare systems. To fulfil the United Nations Sustainable Development Goals, the healthcare sector must undergo a transformation towards more eco-friendly practices, which will also affect clinical decision-making. Net-zero is an internationally agreed goal for avoiding worsening global heating in the second half of the 21st century, as it aims to balance the quantities of greenhouse gases released into and removed from the atmosphere, achieving carbon neutrality. Nevertheless, the operating room (OR) is among the most significant contributors to environmental pollution, with the major carbon hotspots determined by the use of energy, procurement and disposal of consumables and the waste of water. In response to this challenge, leading medical societies, surgical teams, government bodies, and industry stakeholders endorse Green Surgery (GS) by taking steps to address healthcare sustainability and its impact on climate change. Therefore, a movement toward “greening” healthcare, or improving the environmental footprint of healthcare has been built.

## 1. Introduction

Climate change has been identified by the World Health Organization (WHO) as “the single greatest health threat to humanity”. It contributes to the intensification of extreme weather conditions, disruption of global food supplies and the rising incidence of zoonotic diseases, collectively posing profound challenges to human health, clinical practice and the resilience of healthcare systems [1,2]. In April 2024, the European Court of Human Rights delivered a landmark decision affirming that governments have a legal obligation to safeguard their citizens from the risks and consequences of climate change [1]. The significance of environmental sustainability is recognized across all healthcare systems. To fulfil the United Nations Sustainable Development Goals set forth in the 2030 Agenda, the healthcare sector must undergo a transformation towards eco-friendlier practices, which will also affect clinical decision-making [3]. Net-zero is an internationally agreed goal for avoiding worsening global heating in the second half of the 21st century, as it aims to balance the quantities of greenhouse gases released into and removed from the atmosphere, achieving carbon neutrality [4].

Paradoxically, while healthcare aims to protect human health, it is responsible for 4.4% of global carbon emissions, with the top three healthcare emitters being the United States, China, and the European Union [5]. More specifically, the operating room (OR) remains among the most significant contributors to environmental pollution in the context of healthcare provision, with the major carbon hotspots determined by their use of energy, procurement, disposal of consumables and the waste of water [6]. In response to this challenge, leading medical societies, surgical teams (surgeons, anesthetists, nurses, and operating department staff), government bodies, and industry stakeholders endorse Green Surgery (GS) by taking steps to address healthcare sustainability and its impact on climate change [1,7]. Therefore, a movement toward “greening” healthcare, or improving the environmental footprint of healthcare is underway. Individual hospital-based initiatives have been reported over the past decade, with strategies to successfully reduce medical waste in ORs while at the same time not increasing operating costs [8,9]. To investigate this subject, we conducted a review of the literature about sustainability in the OR, with the aim of exploring the existing knowledge and identifying prevailing challenges in the field.

## 2. Historical Context and Definition

During the past 50 years, the healthcare industry has undergone tremendous changes in the types of products it consumes and the waste it produces [8]. Historically, minimal regulation governed medical waste, as in-hospital incineration was ordinary [8]. However, the emergence of blood-borne pathogens such as HIV and hepatitis led to heightened regulatory scrutiny. The introduction of universal precautions in 1987 and the Medical Waste Tracking Act of 1988 reclassified most clinical waste as potentially infectious, catalyzing a surge in regulated “red bag” disposal. Although intended for items visibly contaminated with bodily fluids (e.g., blood, cerebrospinal fluid, amniotic fluid), red bag usage is frequently excessive due to poor segregation practices and operational convenience. Consequently, vast amounts of non-infectious waste have been misclassified and incinerated, escalating environmental and financial costs [8]. Additionally, the adoption of modern surgical systems, energy-intensive and reliant on nonrenewable resources, has further compounded surgery’s ecological footprint [10].

It has been gradually evaluated that the environmentally harmful practices of the healthcare industry represent a very real threat not only to the longevity of the healthcare system, but also to our planet and human health [11]. Early surgical sustainability efforts centred on waste reduction and the reuse of selected instruments [10]. The practice of medical device reprocessing—encompassing cleaning, sterilization and repackaging—emerged as a strategic response to the growing environmental impact of surgery. These foundational measures catalyzed the development of what would later be termed “Green Surgery”, as clinicians increasingly advocated for systemic and environmentally sustainable operative practices [10].

An important part of GS’s modern evolution has been the establishment of reports, conferences and initiatives that have already played a pivotal role in advancing the agenda of sustainable surgery, providing frameworks and inspiration for healthcare professionals worldwide to integrate environmental considerations into surgical care. A selection of representative initiatives is listed to highlight their contribution to advancing environmentally sustainable practices within the surgical field. Firstly, the Green Surgery Challenge (2021) was created as an opportunity to share and promote ways of practicing surgery that are less harmful to the environment and build social sustainability, through team-mentoring from specialists, designing and implementing original projects, all by using sustainable quality improvement (SusQI) methodology [12]. Secondly, the Research for Greener Surgery Conference (2023), organized by the NIHR Global Surgery Unit, was the first-ever conference focusing on research around decarbonizing operating theatres and aimed to engage clinical and non-clinical staff, members of the industry and other stakeholders and to create discussion about ideas, research and innovation in sustainable surgery, with an emphasis on ongoing research projects and training opportunities [13]. Thirdly, the Green Surgery Report, released in 2023, is the first survey of its kind by any medical specialty and is badged by many organizations [9]. It provides current evidence and case studies on how to minimize the environmental harm of surgical care, through better design and use of operating theatres, among others. It is intended primarily as a tool for surgical teams but also outlines actions and recommendations for other stakeholders [9].

According to the Webster Dictionary, the term “environmentally sustainable” corresponds to “goods and services, laws and policies that claim reduced, minimal or no harm on the environment” [14]. It is also assessed as environment-friendly, eco-friendly, nature-friendly and green processes. Applied to the health system, the WHO has defined it as a process that “could improve, maintain or restore health, while minimizing negative impacts on the environment”. On a broader scale, healthcare must, not only eliminate waste, but also reach sustainable development, which is defined as “meeting the needs of the present without compromising the ability of future generations to deal with their own needs” [14]. Moreover, the GS context includes a rising awareness of the healthcare industry’s role in global pollution and the need for more sustainable therapies [10]. GS aims to optimize the carbon footprint of care without affecting surgical and non-surgical outcomes [2]. Furthermore, “greening initiatives” include not only reducing, recycling and reusing, but also rethinking and researching, as well as implementing novel technology and smarter architectural designs [11]. Therefore, GS has grown into a multidisciplinary approach that considers the technical elements of surgical treatments and the broader ethical and environmental implications of medical practice [10].

## 3. Aim of Review

To investigate the subject of the climate impact caused by surgery, we conducted a narrative review of the literature about sustainability in the OR, with the aim of exploring the existing knowledge and identifying prevailing challenges in the field.

## 4. Materials and Methods

This study was conducted as a narrative literature review performed via a structured literature search designed to identify the most relevant evidence regarding Green Surgery and environmentally sustainable surgical practice. A combined automated and manual database search of the literature, more akin to a systematic review, was performed using various electronic search engines (Medline PubMed, Scopus, Ovid and Cochrane Library), reaching up to 21 May 2025 so as to ensure completeness. Additional articles were identified through manual screening of reference lists from selected publications. Initially, the study abstracts were screened by two of the authors for relevant keywords which were pre-defined and included the terms: “green surgery,” “sustainable surgery,” “environmental sustainability,” “carbon footprint,” “operating room waste,” “healthcare sustainability,” “surgical waste management,” and “de-carbonization in healthcare.” Boolean operators (AND, OR) were used to optimize the search. Two authors acting as reviewers independently screened titles and abstracts for relevance. Full-text articles were subsequently assessed for eligibility based on the predefined inclusion and exclusion criteria.

Any disagreements between the reviewers regarding study inclusion were resolved through discussion and consensus. Reviewer agreement was not formally quantified (e.g., by percentage agreement or Cohen’s kappa), as this narrative review employed a consensus-based screening process. The final selection of articles was based on their relevance to the objectives of this review, with emphasis placed on studies providing original data, comprehensive reviews, guideline recommendations, or practical experience related to environmentally sustainable surgical practice. Third-party adjudication was therefore not required. Duplicate records identified across PubMed, Scopus, Ovid, and Cochrane were removed before article screening.

Articles were included if they addressed environmental sustainability within surgical practice or perioperative care, reported interventions, strategies, policies, or outcomes related to reducing environmental impact in surgery and were published in English in peer-reviewed journals. The following were excluded: conference abstracts without full-text availability, editorials lacking substantive discussion of sustainability measures, studies unrelated to surgical or perioperative settings and non-English publications as a potential source of language bias. Given the emerging nature of environmentally sustainable surgery, evidence was drawn from a range of source types, including peer-reviewed original studies, review articles, professional society guidelines, institutional reports, and selected organizational publications. Priority was given to peer-reviewed literature whenever available. Non-peer-reviewed sources were included only when they originated from recognized healthcare organizations, professional societies, academic institutions, or governmental bodies and provided relevant information, recommendations, or examples of sustainable surgical practice. All sources were assessed for their relevance, credibility, and contribution to the objectives of the review. During the narrative synthesis, empirical evidence derived from peer-reviewed original studies, Life Cycle Assessment analyses, and systematic or narrative reviews formed the primary basis for evaluating the effectiveness of sustainable surgical interventions. Institutional reports, professional society documents, implementation case studies, and organizational publications were incorporated primarily to illustrate current practices, implementation strategies, and real-world examples of Green Surgery initiatives. These sources were used to provide contextual information and were interpreted in conjunction with the available peer-reviewed evidence rather than as standalone evidence supporting clinical or environmental effectiveness.

This manuscript was designed as a narrative review supported by a structured literature search rather than as a formal systematic review. Consequently, the search strategy was intended to identify the most relevant and representative evidence on Green Surgery rather than to exhaustively capture all available publications. Accordingly, a formal PRISMA study-selection process was not applied, as the manuscript was not designed as a systematic review. Nevertheless, predefined search terms, multiple databases, independent screening by two reviewers, and explicit inclusion and exclusion criteria were used to enhance the transparency and reproducibility of the review methodology. As the objective of the structured literature search was to identify the most relevant evidence to inform this narrative review rather than to perform a comprehensive systematic evidence synthesis, quantitative reporting of the search yield was not undertaken. Consistent with the narrative design of this review, no formal methodological quality assessment or risk-of-bias tool was applied to the included literature. The thematic organization of the review was predefined by the authors based on the objectives of the study, with sections addressing the historical development of Green Surgery, current evidence, clinical feasibility, economic considerations, and future recommendations. Within this framework, evidence identified through the structured literature search was narratively synthesized and incorporated into the section that most appropriately reflected its principal focus. This approach enabled a coherent presentation of the available literature while accommodating the heterogeneous nature of the evidence base.

## 5. Feasibility of Sustainable Surgery

The following examples illustrate representative institutional initiatives that demonstrate the practical implementation of Green Surgery principles. These examples are intended to provide real-world context for current sustainability practices and should be interpreted alongside the broader peer-reviewed evidence summarized elsewhere in this review.

The NHS has committed to achieving net carbon zero by 2040. This ambitious target cannot be realized without significantly reducing the environmental impact of surgical practice. The Research for Greener Surgery Conference 2023 encouraged experts to consider how healthcare providers can de-carbonize surgery while maintaining the highest standards of patient care. Surgeons and academics from around the world gathered on the Birmingham campus in late 2023 to take into consideration research on achieving net-zero emissions in operating theatres [15]. This groundbreaking procedure, performed first at Solihull Hospital, integrated a variety of evidence-based sustainability strategies, incorporating measures like using reusable surgical materials, minimizing energy consumption and offsetting remaining emissions through verified projects. The success of this operation demonstrates the feasibility of reducing the environmental impact of surgical practices without compromising patient care [16].

Leeds Teaching Hospitals NHS Trust is recognized as one of the UK’s most progressive healthcare institutions in advancing sustainable surgical practices, contributing significantly to the NHS’s broader ambition of achieving net-zero carbon emissions by 2040 [17]. In a significant effort to reduce greenhouse gas emissions associated with surgical procedures, the Trust piloted the use of the Retractor for Abdominal Insufflation-less Surgery (RAIS) device during appendectomy procedures. This innovative approach eliminates the requirement for carbon dioxide insufflation, commonly employed in conventional laparoscopic techniques, by mechanically elevating the abdominal wall to create a safe and effective operative field. The RAIS device significantly reduces the carbon footprint of minimally invasive surgery, marking a substantial advancement in the development of gasless laparoscopic techniques [17].

UCSF (University of California, San Francisco) Medical Center was among the first academic medical institutions to completely phase out the use of Desflurane, one of the most environmentally harmful inhaled anesthetics. The institution now prioritizes the use of Sevoflurane and Total Intravenous Anesthesia (TIVA), achieving a hospital-wide reduction in anesthetic-related greenhouse gas emissions by more than 80%. In parallel, reusable surgical instruments and re-processable devices have become standard practice at UCSF. The institution also collaborates with environmentally responsible suppliers to minimize the life-cycle carbon footprint of surgical materials [18].

Similarly, Cleveland Clinic has gained international recognition for integrating sustainability principles into surgical practices and broader healthcare delivery. As a leading multi-specialty academic medical centre, it has introduced several environmentally conscious measures in the operating theatre. These include a comprehensive waste segregation system using colour-coded bins to separate clean plastics, blue wrap and recyclable medical supplies, as well as installed energy-efficient LED lighting and occupancy sensors in operating theatres. These initiatives have significantly reduced regulated medical waste, cutting disposal costs and associated emissions. Notably, a significantly reduced portion of energy consumption in the operating suite stems from heating, ventilation, and air conditioning (HVAC) systems, which are required not only for maintaining thermal comfort but also for ensuring the sterile environment required for surgical safety [19].

In Europe, Valld’Hebron University Hospital in Spain stands at the forefront of sustainable surgical innovation, demonstrating how advanced technologies can support environmental responsibility in high-acuity healthcare settings. As one of Europe’s largest and technologically most advanced hospitals, Valld’Hebron has implemented intelligent climate-control systems in its operating theatres, leveraging real-time patient-flow data to dynamically regulate temperature and ventilation. This automated approach ensures optimal clinical conditions while substantially reducing unnecessary energy consumption [20]. By aligning operational efficiency with environmental stewardship, the hospital illustrates that progress toward carbon neutrality can coexist with the delivery of world-class surgical care. This model provides a compelling precedent for global health systems seeking to balance clinical excellence with urgent environmental imperatives [20].

Finally, Karolinska University Hospital in Sweden is a widely recognized leader in integrating sustainability into surgical care, pioneering innovative strategies to reduce the environmental footprint of its operating theatres [21]. The hospital has focused on optimizing energy consumption by upgrading to energy-efficient lighting and advanced ventilation technologies designed specifically for surgical environments. Additionally, Karolinska has adopted the use of low-impact anesthetic gases, significantly mitigating greenhouse gas emissions associated with traditional anesthetic agents. The institution has also developed a comprehensive waste management programme focused on effective waste segregation, recycling, and minimizing reliance on single-use materials, contributing to notable reductions in regulated medical waste [21]. By demonstrating that high-quality surgical care can coexist with ambitious carbon reduction targets, Karolinska provides a compelling model for healthcare institutions worldwide striving to balance patient safety with urgent ecological responsibilities. This integrated strategy highlights that sustainable surgical practice is not only achievable but also essential for the future of environmentally responsible healthcare delivery.

Although these initiatives demonstrate the feasibility of implementing Green Surgery, the magnitude of their environmental benefits varies considerably. Representative quantitative findings from the literature are summarized in Table 1.

## 6. Critical Appraisal of Current Evidence and Remaining Challenges

Although numerous sustainability initiatives have been proposed for operating theatres, the strength of evidence supporting individual interventions varies considerably. Most published studies are observational, single-centre quality improvement projects, modelling studies, Life Cycle Assessment (LCA) analyses or review articles, while randomized studies and multicentre prospective evaluations remain scarce [22,23,24,25,26,27]. Consequently, recommendations should be interpreted according to evidence quality rather than reported environmental benefit or the magnitude of reported carbon reductions [22,26,27].

Among available interventions, reducing the environmental impact of anesthesia (desflurane elimination, nitrous oxide reduction and low-flow anesthesia) has the strongest and most consistent evidence, followed by reusable instruments and optimized procurement [22,23,24,25,26]. Waste segregation is highly feasible but contributes less to overall carbon reduction than procurement or anesthetic changes [22]. Energy-saving infrastructure demonstrates considerable promise but is currently supported mainly by hospital implementation studies [22,23,24,25].

To facilitate comparison between the principal sustainability interventions, Table 2 summarizes the current evidence according to the magnitude of carbon reduction, quality of supporting evidence, implementation challenges, patient safety considerations, and economic implications. This comparison highlights that not all interventions provide equivalent environmental benefits or have the same level of supporting evidence, emphasizing the need to prioritize strategies that offer the greatest environmental impact while maintaining clinical effectiveness.

Implementation barriers include infection prevention concerns, financial constraints, lack of institutional leadership, procurement challenges, limited education and the absence of standardized sustainability metrics [25]. Sustainable interventions should always preserve patient safety and clinical effectiveness [25]. Reusable devices require validated sterilization processes, whereas robotic surgery may improve selected outcomes despite higher environmental impacts [25,28]. Important knowledge gaps include heterogeneous outcome reporting, limited use of Life Cycle Assessment and the predominance of observational studies. Future multicentre prospective studies are needed to explore the concepts further.

## 7. Economic Considerations and Cost-Effectiveness of Green Surgery

Although the primary objective of Green Surgery is to reduce the environmental impact of surgical care, the successful implementation of sustainable practices also depends on their economic feasibility. Environmental benefits alone are unlikely to drive widespread adoption unless interventions are practical, affordable and compatible with the financial realities of modern healthcare systems. Consequently, sustainability initiatives should be evaluated not only according to their environmental performance but also according to their economic impact and long-term value for healthcare institutions [7,9,25].

The financial implications of Green Surgery vary considerably depending on the intervention. Some initiatives, including energy-efficient operating theatre infrastructure, upgraded ventilation systems and reusable surgical equipment programmes, require initial capital investment and organizational commitment before financial benefits can be realized [22,27]. These upfront costs may represent an important barrier for institutions with limited financial resources and have been identified among the principal challenges limiting the broader implementation of sustainable surgical practices [25].

Nevertheless, increasing evidence suggests that several sustainability interventions may generate long-term financial savings while simultaneously reducing environmental impact. Life Cycle Assessment studies comparing reusable and single-use surgical equipment have demonstrated that reusable devices are frequently associated with lower life-cycle environmental impact and lower overall financial costs after accounting for acquisition, sterilization, maintenance and disposal expenses [24,26]. Similarly, interventions targeting anesthetic practice, including the elimination of desflurane and the adoption of low-flow anesthesia, have been shown to reduce greenhouse gas emissions while decreasing the consumption of volatile anesthetic agents, thereby lowering operating costs [22,23]. Furthermore, improved waste segregation decreases the volume of regulated medical waste requiring expensive disposal and represents a simple intervention that can reduce both environmental burden and waste-management expenditure [8,22].

Economic evaluation should therefore accompany environmental assessment when prioritizing sustainable surgical interventions. While Life Cycle Assessment (LCA) remains the reference methodology for quantifying environmental impact, incorporating life-cycle financial analyses enables a more comprehensive evaluation of healthcare technologies by considering procurement, maintenance, sterilization, energy consumption and disposal costs throughout the product lifespan [22,23,26]. Such integrated assessments may assist hospitals in identifying interventions that provide the greatest environmental benefit while remaining economically sustainable.

Despite encouraging findings, economic evidence in Green Surgery remains relatively limited. Most published studies are observational investigations, single-centre quality improvement projects or Life Cycle Assessment analyses, whereas standardized cost-effectiveness evaluations and prospective multicentre studies remain scarce [22,24,27]. Consequently, future research should incorporate both environmental and economic outcome measures to facilitate evidence-based decision-making and allow meaningful comparison between alternative sustainability strategies.

## 8. Future Recommendations

Considering these data, it is evident that surgeons’ point of view worldwide is maturing and shifting towards the necessity for a revolution in the way of practically thinking about delivery of care, addressing clinical outcomes and environmental impact [2]. Following greener practices, a variety of ideas and opinions have emerged. It is common knowledge that surgeons play a pivotal role in global health. To approach sustainability as a matter of environmental, economic and ethical priority, surgical practice should gradually change the use of minimally invasive (MIS) technologies such as laparoscopy and robotic surgery, which will need to be critically appraised from a “greener” perspective [5].

Since the introduction of MIS techniques, the operating room environment has become the top producer of biohazard hospital waste and energy expenditure, with the estimated carbon footprint of a single operation being equivalent to driving an average gas-powered car for more than 2000 miles. Reliance on disposable equipment and instruments needs to be challenged, together with the use of excess water in the OR and excess waste generation [5]. Every relevant individual (including surgeons, staff in sterile service departments and perioperative staff) should be encouraged to look after their equipment and actively monitor for and promptly repair relevant defects [29].

Although minimally invasive surgery (MIS) is generally associated with shorter hospital stay, reduced postoperative pain, lower wound complication rates and faster recovery than open surgery, its environmental impact varies considerably depending on the technology employed [7,22,23].

In particular, robotic surgery has become an area of growing interest because of its increasing adoption worldwide and its potential environmental implications. While robotic platforms provide important clinical advantages in selected procedures, including improved dexterity, enhanced three-dimensional visualization, tremor filtration and greater precision during complex operations, these benefits must also be evaluated alongside their environmental footprint [7,22,27].

Current evidence suggests that robotic surgery is generally associated with higher greenhouse gas emissions than conventional laparoscopy. Comparative Life Cycle Assessment (LCA) studies have reported increases of up to 43% in carbon dioxide equivalent (CO_2_e) emissions and approximately 24% greater waste generation, primarily because of increased energy consumption, larger numbers of disposable instruments, complex robotic accessories and more demanding sterilization requirements [22,30].

Furthermore, robotic procedures frequently require dedicated single-use drapes, trocar systems, stapling devices and limited-use instruments that are discarded after a predetermined number of uses, substantially increasing procurement-related emissions, which represent one of the largest contributors to the carbon footprint of modern operating theatres [22,23,27].

Beyond the operating room itself, robotic surgery also carries additional environmental burdens throughout the supply chain. Manufacturing robotic systems requires considerable quantities of raw materials, electronic components and specialized metals, while transportation, maintenance and servicing further contribute to life-cycle emissions. In addition, robotic systems require larger operating rooms, prolonged equipment setup and higher electricity demand during both active operation and standby periods [22,24,27].

Nevertheless, the relationship between robotic surgery and sustainability is not entirely one-sided. Improved surgical precision may reduce complications, decrease conversion to open surgery and shorten hospital stay in selected patient populations. These downstream clinical benefits may partially offset the increased environmental burden associated with the procedure itself, although robust evidence quantifying these indirect environmental effects remains limited [7,26,27].

Furthermore, manufacturers have recently introduced reusable or hybrid robotic instruments and recycling programmes for selected consumables, demonstrating that technological innovation may progressively reduce the environmental impact of robotic platforms [22,26,30].

Therefore, rather than considering robotic surgery as inherently environmentally unfavourable, future research should focus on identifying those procedures in which its clinical benefits justify the additional environmental cost. Standardized Life Cycle Assessment methodologies incorporating both direct procedural emissions and downstream healthcare utilization will be essential to determine the true sustainability profile of robotic-assisted surgery [22,24,27]. Such evidence will enable surgeons, healthcare systems and policymakers to balance environmental responsibility with patient safety, clinical effectiveness and healthcare value [7,22,26].

Although robotic-assisted surgery offers important clinical advantages in selected procedures, current evidence indicates that its environmental impact is generally greater than that of conventional laparoscopy because of higher energy requirements, increased reliance on disposable devices, more complex supply chain logistics, and greater procurement-related emissions. Nevertheless, the magnitude of these differences varies according to procedure type, institutional practices, and methodological assumptions used in Life Cycle Assessment studies. To provide an evidence-based overview of these considerations, Table 3 compares laparoscopic and robotic surgery across key sustainability domains, including carbon emissions, waste generation, resource utilization, costs, and clinical implications.

Overall, the available evidence suggests that laparoscopic surgery currently demonstrates a more favourable environmental profile than robotic-assisted surgery in most Life Cycle Assessments. However, robotic surgery may provide important patient and surgeon benefits in selected complex procedures that are not captured by carbon footprint analyses alone. Consequently, future sustainability evaluations should integrate environmental outcomes with clinical effectiveness, patient-centred outcomes, and cost-effectiveness to identify procedures in which robotic-assisted surgery provides sufficient value to justify its additional environmental burden.

**Figure 1:**
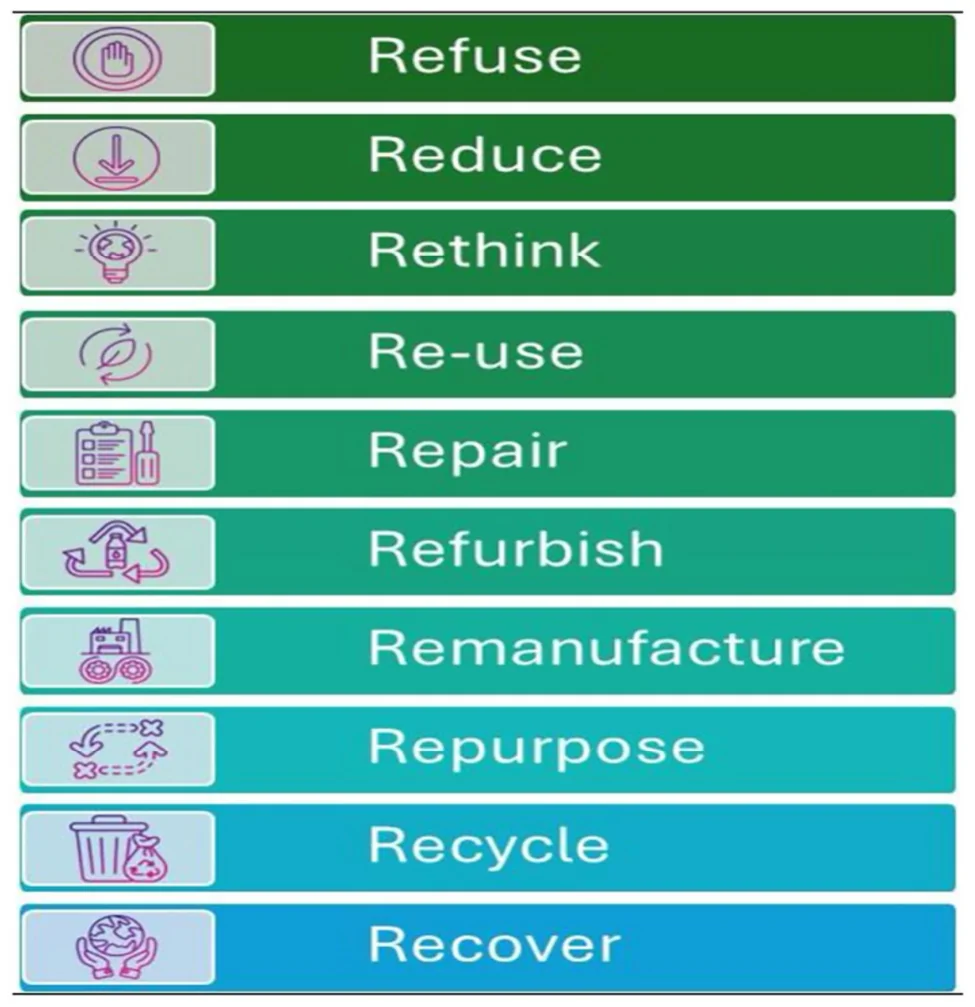
The “10R Model of Circular Economy” provides a structured and practical framework for classifying and evaluating the environmental impact of sustainable surgical interventions.

Hospital-level initiatives remain at the basis of every sustainable intervention towards environmentally friendly OR activity. Multidisciplinary collaborations in every hospital should include professionals from across the perioperative pathway: surgeons, anesthesiologists, surgical nurses, infection control personnel, as well as sterile processing teams, waste management services, supply chain officers and hospital executives. The established objective is to design and implement targeted interventions to reduce emissions and waste within their sphere of influence. Each multidisciplinary “Green Surgical Team” could design its specific institutional policy based on specific interventions in the operating room with measurable impacts based on the so-called “10R model of circular economy” (Figure 1) [28]. This model, which has been endorsed by the joint Sustainability in Surgical Practice (SSP) Society of American Gastrointestinal and Endoscopic Surgeons (SAGES) and the European Association for Endoscopic Surgery (EAES) Taskforce, is an effective approach to address surgical sustainability and could be implemented by every institutional “Green Surgical Team” as a guide to design its own energy-reducing strategy.

In practice, the 10R model provides a simple framework for selecting sustainability initiatives according to local priorities. For example, hospitals may reduce unnecessary disposable items, repair reusable equipment when feasible, optimize recycling of non-infectious waste, and prioritize procurement strategies that extend product lifespan. Rather than implementing isolated interventions, the framework encourages a systematic approach to continuous environmental improvement while maintaining patient safety.

**Figure 2:**
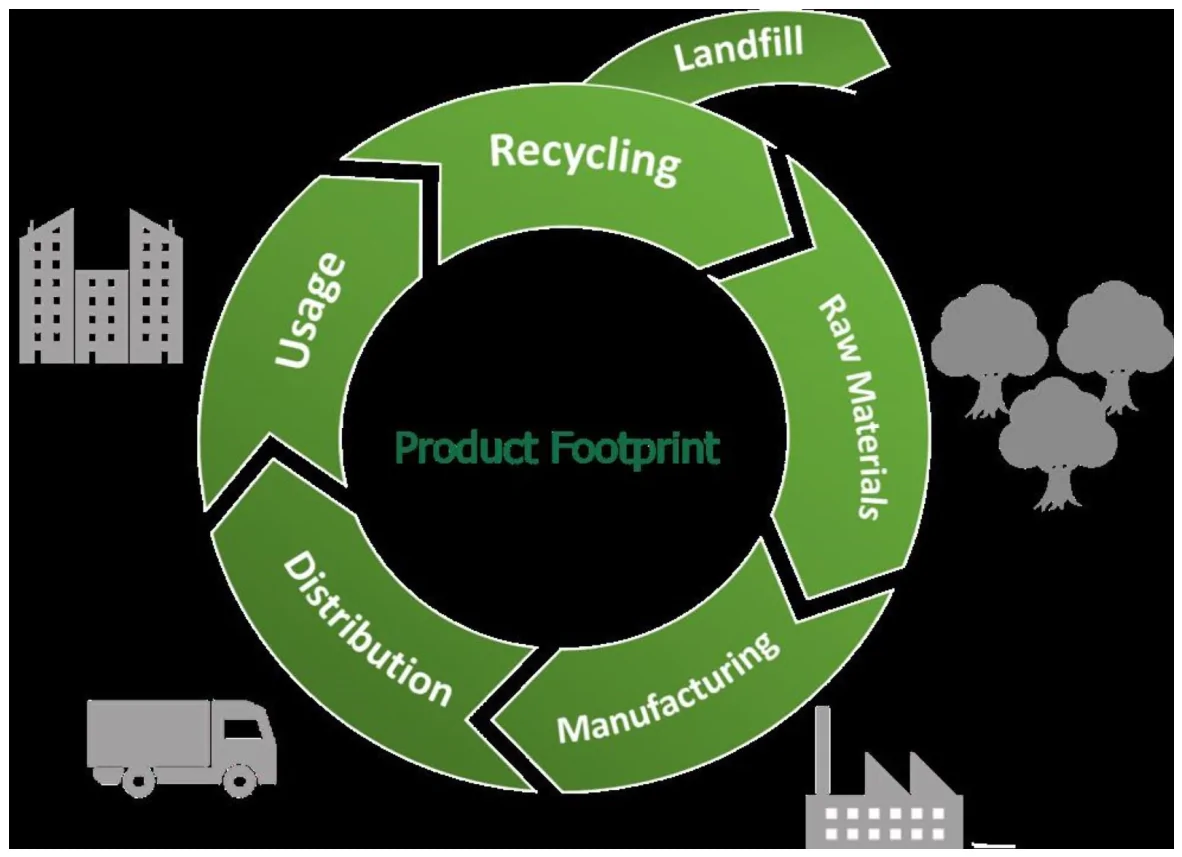
Life Cycle Assessment (LCA) is a rigorous methodology for studying the environmental impact of a product or process. It considers both the upstream and disposal of products to capture all inputs and outputs.

A widely accepted interpretation of outcomes remains at the cornerstone of any new initiative in the future, as significant heterogeneity in study design and outcome measures exists across included studies. Life Cycle Assessment (LCA) is a rigorous methodology for studying the environmental impact of a product or process. It considers both the upstream processes and disposal of products to capture all inputs and outputs (Figure 2) [29]. Even if LCA remains the gold standard in sustainability research, only a minority of studies have utilized this approach so far [25]. It could be associated with time and resource limitations, which prevent its wider use [31]. It may be more feasible to measure climate impact using non-LCA studies with a selected but important group of outcomes, including resource utilization such as the consumption of energy or water associated with a particular product, process, or service, or via economic analysis of the cost associated with implementing a sustainable initiative or solution. Non-LCA studies evaluating climate impact or resource use may apply the 10R model (see Figure 1) of circular economy, which provides a structured and practical framework for classifying and measuring the environmental impact of sustainable interventions [1].

Life Cycle Assessment is particularly useful when comparing alternative surgical products or techniques. For example, it can be applied to evaluate reusable versus single-use instruments or different anesthetic approaches by considering emissions generated during manufacturing, transportation, use, sterilization, and disposal. This enables hospitals to prioritize interventions with the greatest overall environmental benefit.

At the same time, national and international organizations should establish ambitious sustainability goals, as it is up to individual institutions to discover innovative ways to meet those targets within the limits of their own resources and circumstances. Gaining insight into surgeons’ attitudes, knowledge gaps and ability to adopt sustainable practices is a crucial first step in identifying both barriers and opportunities for broader progress. Moving forward, surgical societies should focus on creating educational initiatives and providing practical guidance to support the gradual adoption of environmentally sustainable practices in surgery [31,32,33]. Knowledge about the various elements of “Carbon Hotspots” in the OR can empower surgical teams to modify behaviour and make incremental changes to reduce the carbon footprint without adverse consequences on patient care. This entails updating the members with the relative contribution of electricity, anesthetic agents, procedural approach, reusable vs. disposable supplies and instruments and surgical waste to the overall procedural footprint [7].

As a next step, institutions will need to find innovative ways to meet sustainability targets within their specific limitations, taking into account the values and preferences of their surgeons. Finally, on the basis of definite global resources, we need to apply circular economy principles whereby we maximize resource use through maintenance, repair and recycling in order to extend the lifespan of our consumables and capital goods. A large proportion of waste generated in theatres is potentially recyclable, and more should be accomplished in this direction [25]. In addition, healthcare facilities and data centres that support AI systems should implement green computing strategies to reduce their environmental impact. This involves utilizing energy-efficient hardware, optimizing software performance and designing sustainable infrastructure. Techniques like dynamic voltage and frequency scaling can help lower energy usage during times of reduced activity. Additionally, incorporating renewable energy sources, such as solar and wind power, into their energy supply can significantly limit carbon emissions [31].

## 9. Conclusions

Green Surgery has emerged as a necessary response to the environmental challenges posed by modern healthcare practice. Operating rooms represent a major source of healthcare-related carbon emissions, waste generation, and resource consumption. Evidence suggests that sustainable practices, including waste reduction, reuse of equipment, energy-efficient technologies, and environmentally conscious procurement, can significantly reduce the environmental footprint of surgical care without compromising patient outcomes and high standards. Achieving meaningful progress will require multidisciplinary collaboration, institutional commitment, standardized sustainability metrics, and ongoing education. Nevertheless, the main barriers to relevant implementation include infection control and safety strategies, financial and resource constraints and leadership and policy gaps. Furthermore, there is a strong perception that procuring reusable items or implementing energy-saving infrastructure increases treatment and upfront costs. Current evidence indicates that interventions targeting anesthetic gases, reusable surgical equipment and optimized resource utilization possess the strongest environmental evidence and can substantially reduce operating-room carbon emissions without compromising patient outcomes. However, much of the literature remains based on observational studies, quality improvement projects and life-cycle modelling rather than high-quality prospective investigations. Future implementation strategies should therefore integrate environmental and economic evaluations to identify interventions that provide the greatest clinical, environmental and financial value. Standardized reporting, multicentre implementation studies and economic evaluations are required before many interventions can be universally recommended.

This review has several limitations. As a narrative review supported by a structured literature search, it was not intended to provide an exhaustive systematic synthesis of the available evidence. Accordingly, no formal methodological quality assessment or risk-of-bias evaluation of the included studies was performed, which should be considered when interpreting the findings. In addition, only English-language publications were included, which may have introduced language bias and resulted in the exclusion of relevant reports or experiences from non-English-speaking countries. Given the global nature of sustainability initiatives in surgery, future reviews incorporating multilingual literature and formal quality appraisal may provide a broader and more rigorous synthesis of the available evidence.

## Figures and Tables

**Table 1:** Representative quantitative environmental benefits reported for major Green Surgery interventions.

Intervention	Quantitative Finding	CO_2_e/Environmental Benefit	Evidence	References
Desflurane elimination	Hospital phase-out	≈70–80% reduction in anaesthetic GHG emissions	High	Refs. [18,22,23]
Low-flow anesthesia/TIVA	Reduced fresh gas flow	20–50% lower volatile anesthetic emissions	Moderate–High	[22,23]
Reusable instruments	Reusable vs. disposable	30–80% lower life-cycle CO_2_e	Moderate	[23,24]
Waste segregation	Improved segregation	20–50% less regulated waste; modest CO_2_ benefit	Moderate	[8,25]
HVAC optimization	Smart ventilation	10–70% energy savings	Moderate	[20]
Robotic vs. laparoscopic	Comparative studies	Up to 43% higher GHG emissions; 24% more waste	Moderate	

**Table 2:** Comparative ranking of sustainability interventions.

Intervention	Carbon Reduction	Evidence Quality	Implementation	Safety	Economic Implications
Desflurane elimination	Very High	High	Easy	No adverse effect	Lower anesthetic expenditure; minimal implementation cost
Reusable devices	High	Moderate–High	Moderate	Validated sterilization	Higher initial investment; lower life-cycle cost
Low-flow anesthesia	High	High	Easy	No adverse effect	Reduced volatile anesthetic consumption and operating costs
Energy-efficient infrastructure	Moderate–High	Moderate	Complex	Maintain standards	High upfront capital cost with long-term energy savings
Waste segregation	Low–Moderate	Moderate	Easy	None	Lower regulated waste disposal costs
Recycling	Low	Low	Easy	None	Variable savings depending on local recycling infrastructure

**Table 3:** Comparison of laparoscopic and robotic surgery from a sustainability perspective.

Parameter	Laparoscopic Surgery	Robotic Surgery
Operating-room energy consumption	Lower	Higher
Disposable instrument use	Moderate	High
Reusable instrument availability	Greater	Limited but increasing
Procurement-related emissions	Lower	Higher
Carbon footprint (CO_2_e)	Lower	Up to 43% higher
Waste generation	Lower	Up to 24% higher
Supplychain complexity	Moderate	High
Equipment maintenance	Limited	Extensive
Upfront capital cost	Lower	Very high
Clinical advantages	Excellent for many procedures	Superior dexterity, ergonomics, visualization in selected procedures
Overall sustainability	Generally favourable	Procedure-dependent; requires optimization

## Data Availability

No new data were created or analyzed in this study. Data sharing is not applicable to this article.
